# Association Between the Quality of Contraceptive Counseling and Method Continuation: Findings From a Prospective Cohort Study in Social Franchise Clinics in Pakistan and Uganda

**DOI:** 10.9745/GHSP-D-18-00407

**Published:** 2019-03-22

**Authors:** Nirali M. Chakraborty, Karen Chang, Benjamin Bellows, Karen A. Grépin, Waqas Hameed, Amanda Kalamar, Xaher Gul, Lynn Atuyambe, Dominic Montagu

**Affiliations:** a Metrics for Management, Baltimore, MD, USA.; b Population Council, New York, NY, USA.; c Wilfrid Laurier University, Waterloo, Canada.; d Aga Khan University, Karachi, Pakistan.; e Population Services International, Washington, DC, USA.; f Marie Stopes Society, Karachi, Pakistan.; g Makerere University, Kampala Uganda.

## Abstract

Quality of family planning counseling is likely associated with whether or not women continue to use the same contraceptive method over time. The Method Information Index (MII) is a widely available measure of contraceptive counseling quality but little is known about its association with rates of method continuation. The index ranges from 0 to 3 based on a client's answer to whether she was told about other methods, potential side effects with her chosen method, and what to do if she experienced side effects. Using data from a prospective cohort study of 1,998 social franchise clients in Pakistan and Uganda, we investigated the relationship between reported baseline MII and the risk of method continuation over 12 months using survival analysis and Cox proportional hazard models. At baseline, about 65% of women in Pakistan and 73% of women in Uganda reported receiving information about all 3 MII aspects. In Pakistan, 59.4% of the 165 women who stopped using their modern method did so while still in need of contraception. In Uganda, of the 77 women who stopped modern method use, 64.9% discontinued while in need. Despite important differences in the demographics and method mix between the 2 countries, we found similar associations between baseline MII and discontinuation: in both countries as the MII score increased, the risk of discontinuation while in need decreased. In Pakistan, the risk of contraceptive discontinuation was 64% lower (crude hazard ratio [HR_crude_]=0.36; *P*=.03), and 72% lower (HR_crude_=0.28; *P*=.007), among women who were told about any 2, or any 3 aspects of MII, respectively. After adjusting for additional covariates, only the difference in the risk of contraceptive discontinuation between MII=3 and MII=0 remained statistically significant (HR_adj_=0.35; *P*=0.04). In Uganda, women who reported being informed about all aspects of MII were 80% less likely to discontinue while in need (HR_adj_=0.20; *P*<.001), women informed about any 2 aspects of MII were 90% less likely (HR_adj_=0.10; *P*<.001), and women who were informed about any 1 aspect of MII were 68% less likely (HR_adj_=0.32; *P*<.02) to discontinue contraceptive use while in need as compared to women who reported not being informed about any aspect of MII. Baseline MII scores were positively associated with method continuation rates in our sample of clients from social franchises in both Pakistan and Uganda and could potentially be used as an indicator of contraceptive counseling quality.

## Abstract (precis)

Higher scores on the 3-question Method Information Index (MII)—measuring client-reported receipt of contraceptive information—was associated with continued use of family planning over 12 months. We recommend incorporating use of the MII in routine assessments of family planning service quality.

## BACKGROUND

Quality of care is increasingly recognized as a critical driver of health care seeking, use, and outcomes.^1^ Family planning quality has been integral to the vision of the Family Planning 2020 (FP2020) global partnership from its inception in 2012. The partnership's goal is to reach 120 million additional women and girls with modern contraceptives by 2020 by expanding access to contraception, assuring method choice, overcoming barriers to use, and improving quality of care.^2^ This goal recognizes that some new modern method users will come from traditional method users switching to modern methods; therefore, some substitution of method type may result with regard to total use.^2^

In pursuit of this goal, FP2020 has increased its attention on contraceptive discontinuation.^3^ Method discontinuation, for reasons other than a reduced need for contraception, returns women to the pool of those with an unmet need for family planning.^4^^,^^5^ The Demographic and Health Surveys (DHS) defines discontinuation while in need as women who stop contraceptive use without the intent to become pregnant and are at risk of unwanted pregnancy.^6^ In a 2013 analysis of DHS from 34 low- and middle-income countries, Jain et al. found that women who discontinued a modern method while in need accounted for 38% of all women with current unmet need.^4^

While quality has been emphasized in family planning programs for decades based on a widely adopted framework,^7^ and there is some evidence that multidimensional clinical quality is associated with method continuation,^8^ measurement of quality has remained a work-in-progress with many tools but no agreed-on measures, scales, or indicators.^9^

Discontinuation while still in need of contraception, as Jain and others have argued, may be considered an outcome of the quality of family planning services.^4^^,^^5^ Providing women with a choice of contraceptive methods and high-quality counseling are essential components of rights-based family planning.^10^ However, while increased choice has been linked to the increased adoption of family planning,^11^ limited evidence exists on, and it is not yet clear, whether the provision of high-quality counseling is associated with improved outcomes such as continued contraceptive use. A quasi-experimental study by Jain et al. found that provider training to improve information exchange resulted in better counseling received as reported by the client, but there was no significant difference in modern contraceptive continuation.^12^ Léon et al. reported similar results from training with the *Balanced Counseling Strategy*—a toolkit that provides health care providers with information and materials to offer high-quality family planning counseling.^13^^,^^14^ Work on method switching and discontinuation found that clients reporting they had all questions answered by a provider were more likely to switch to another method rather than abandon contraception altogether.^15^ Thus far, limited work has been done to investigate correlations between aspects of quality of counseling (distinct from service readiness) that are associated with sustained contraceptive use.

It is not yet clear whether the provision of high-quality family planning counseling is associated with improved outcomes such as continued contraceptive use.

One important measure of counseling quality is the Method Information Index (MII), which is currently part of the 18 ‘Core Indicators’ tracked by FP2020. The MII is a proxy measure for whether the client received complete counseling, including whether her choice of method was informed. It is a self-reported measure that is used when direct observation of the client-provider interaction is not possible; it captures a woman's recall and understanding of the information exchanged at the time of adoption, in addition to whether the exchange occurred.^16^ While studies have shown that client recall is flawed, and counseling descriptions collected simultaneously from exit interviews and by direct observation may vary significantly,^17^ both collection methods have advantages.^18^ Some argue for the use of client recall because it is less costly to collect and because information that is remembered or ‘received’ may be a better measure of likely impact on behavior and decisions than information ‘provided.’^19^

The Method Information Index is a self-reported proxy measure for whether the client received complete contraceptive counseling.

The DHS has incorporated the 3 questions that comprise the MII in its nationally representative surveys of reproductive-aged women since DHS round IV (1997–2003).^20^ The MII is calculated from responses to 3 questions about the information that contraceptive users received from providers during their family planning visit, consisting of whether they were told about (1) other methods aside from their current method, (2) possible side effects from their current method, and (3) what to do if they experienced side effects.^21^ Users' responses are coded 1 if they answered “yes” or 0 otherwise for each of these questions, and the reported MII score is the percentage of users who responded “yes” to all 3 questions.

The flexibility and versatility of the MII is appealing; in the absence of direct observation of the client-provider interaction, its questions can be asked through exit interviews with family planning clients and mystery clients in addition to being asked of household respondents in the DHS. Given its ubiquity as a measure of system quality at the national level, the MII could be attractive as an indicator of facility-level quality if it could be shown to correlate with family planning outcomes. As yet, limited research has been conducted to test this relationship and to better understand whether MII is related to family planning continuation. There are, moreover, few measures of family planning quality at the facility level that have been shown to be associated with outcomes of any kind.

This study was designed to address this lacuna and to assess the following: Is the MII, as a measure of counseling quality, associated with discontinuation rates at the level of service provision?

This study was designed to assess whether the Method Information Index, as a measure of counseling quality, is associated with discontinuation rates at the service provision level.

We conducted a prospective cohort study in a sample of social franchised family planning facilities in Pakistan and Uganda to understand the relationship between client-reported MII at baseline, a proxy for the quality of counseling received, and 12-month modern method discontinuation while in need. The findings of this study will contribute to the literature on the role of quality of family planning programs in promoting method continuation in low-income country settings.

## METHODS

All data from this study were collected over 12 months in Pakistan and Uganda using similar methodologies. Pakistan and Uganda were selected due to the presence of strong partners and due to the fact that both countries have high rates of unmet need for family planning. In both countries, the partners delivered services through social franchises—networks of private health care providers, linked through a common brand. Typically, in such arrangements, the franchisor provides training, commodities, and quality assurance while the franchisees agree to provide franchised services, be audited, and adhere to price ceilings.^22^ Working with franchises allowed the study to leverage existing administrative and quality assurance systems and to work with a large number of service delivery sites.

This study was conducted in collaboration with Population Services International's (PSI's) Ugandan partner Program for Accessible Health Communication Education (PACE)-Uganda, which operates the ProFam franchise of health clinics throughout the country, and Marie Stopes Society in Pakistan, which operates the Suraj franchise. The ProFam franchise is a network of privately owned health clinics that are located across the country and offer a range of health services, including family planning, HIV, malaria, cervical cancer screening, and maternal health. Clinics are mostly owned by practicing or retired midwives, nurses, nursing assistants, and, in a few cases, medical doctors. The Suraj fractional franchise is focused on family planning services, and the health care providers are primarily midwives.

The study was conducted in social franchise clinics in Pakistan and Uganda.

### Context

Uganda has one of the highest fertility rates in the world (total fertility rate=5.4), and 41% of all births in Uganda are mistimed or unwanted.^23^ Ugandan women have a high need for limiting or spacing pregnancies (67% of married women of reproductive age), but only 35% of married Ugandan women use a modern method of contraception.^23^ According to the 2016 Uganda DHS, Uganda's method mix predominantly consists of short-acting methods, with injectables accounting for more than half of the method mix. The next most frequently used method is implants (18% of modern method mix) while intrauterine devices (IUDs) account for only 4%. However, the situation in Uganda is changing rapidly; data from Performance Monitoring and Accountability 2020 (PMA2020) suggest that the rates of use of implants almost doubled from 15.5% in 2016 to 26.7% in 2018.^24^

In Pakistan, 16% of births in the 5 years preceding the 2012–2013 DHS survey were mistimed or unwanted, and the total fertility rate is 3.8.^25^ Over one-quarter (26.1%) of married women use a modern contraceptive method while 20.1% of married women have an unmet need for family planning. Recent data (2012) from Pakistan indicates that Pakistani women using modern methods are predominantly using female sterilization or condoms (each comprising 35% of modern contraceptive use), with 7% to 11% of contraceptive users using the IUD, pill, or injectable.^25^ Implant availability is increasing, but the method has been available in Pakistan only since 2010; recent data on implant prevalence is not available.^26^

Method discontinuation is an issue both Uganda and Pakistan. In Uganda, 45% of contraceptive users will discontinue within 12 months. Discontinuation rates are highest for pills (67%) and injectables (52%). The most common reason for discontinuation is side effects or health concerns, with more than 1 in 3 users discontinuing for this reason. Of those who discontinued any method and stated wanting another method, just 5% switched to another method within 2 months.^23^ In Pakistan, 37% of women discontinue within 12 months, and the majority of those women discontinue while still in need of contraception (26%). Similar to Uganda, the most common reason for discontinuation in the first year of use is side effects or health concerns, and the rate of switching is relatively low, with 7.6% of contraceptors switching within the first 12 months.^25^ In both countries, more than one-third of current family planning users obtained their method from the private medical sector (35% in Pakistan, 39% in Uganda).^23^^,^^25^ This study was conducted in the private-sector facilities that are affiliated with our study partners.

### Facility Selection, Eligibility, Recruitment, and Follow-Up

In Uganda, we recruited women for this study from high-volume ProFam franchise clinics that provided a full range of modern contraceptive methods. As of July 2016, there were 193 active ProFam clinics operating in Uganda. Of these, we deemed for inclusion 163 clinics (84%) that provided at least 1 of 3 reversible forms of contraceptives (IUDs, implants, or injectables) in the first 6 months of 2016. We restricted the sample to clinics that had at least 28 new family planning clients per month (which was approximately the 50th percentile in the full sample of ProFam clinics) in order to be able to recruit at least 1 new patient per day per clinic. We also limited our sample to clinics in the Central, Southwest, and East regions to minimize data collection costs. However, the excluded regions tended to also have lower patient flows, so this geographic restriction did not reduce the number of high-volume clinics in our sample by very much. We randomly selected 32 clinics from the remaining list of 69 facilities in our sample. Among those selected, 2 clinics were in the process of leaving the ProFam network, so we excluded them from our study, leaving us with 30 clinics in our final sample.

In Pakistan, we used multi-stage sampling to first select 12 districts in 3 provinces of Pakistan (Sindh, Punjab, and Khyber Pakhtoon Khwa). We chose these districts in consideration of project budget and ease of monitoring. Second, all providers who belonged to another project that was closing were dropped from the sampling frame. Finally, 75 Suraj social franchise centers were randomly selected from a total of 81 centers in those provinces in late 2016. The total number of study facilities was chosen based upon a desire to have sufficient heterogeneity in quality while being mindful of feasibility. All Suraj franchises provide condoms, pills, injectables, and IUDs.

In Uganda and Pakistan, PACE-Uganda and Marie Stopes Society, respectively, notified selected facilities about the study and sought their consent to participate in the study. All selected facilities agreed to participate in the study.

Women were eligible to participate in this study if they had received a modern contraceptive method (male or female condom, pill, injectable, implant, IUD, or emergency contraceptive) during the visit in which they were recruited and were either first-time users (reported using contraception for the first time in their life), switching to a different modern method, or lapsed users returning to use (reported not using any contraceptive method in the 3 months prior to the baseline interview). Additionally, to be eligible in Uganda, women must also have provided at least 1 phone number at baseline where they could be reached for follow-up interviews. Women who obtained a resupply of an existing method, received sterilization, or were using non-modern methods, such as withdrawal, were not eligible. All eligible women were asked to provide written informed consent to participate in the study. Exit interviews were conducted in a private setting to ask about their visit and demographics immediately after adopting a modern method during a visit to a social franchise site.

First-time users, method switchers, and lapsed users of reversible contraception were eligible for inclusion in the study.

Study recruitment took place in Pakistan from December 2016 to February 2017, and in Uganda, between February and April 2017. To recruit women, in both countries women exiting a study clinic were screened for eligibility, and if eligible, asked to take an exit survey and also to consent to follow-up at 3, 6, and 12 months after the visit. In Uganda, the women consented for follow-up at 9 months, too. Eligible women who consented to participate were given a short exit survey at the time of recruitment that covered demographics, patient experience, method use, subjective measures of quality and satisfaction, including the 3 items from the MII index, and provider trust (Uganda only). The baseline questionnaire was administered by trained enumerators either inside the clinic or immediately outside of the clinic, depending on the clinic's setup. In both cases, special areas were set aside to conduct the interviews to provide privacy to the women.

In Pakistan, baseline and follow-up data were collected on paper surveys via in-person interviews. Data were double-entered into an EpiData database, and exported to Stata 13 for analysis.^27^^,^^28^ Surveys were conducted in Urdu. Women were requested to provide specific contact details including a phone number, where available, in order to schedule in-person follow-up interviews. At the baseline interview, enumerators discussed how participants wanted to be contacted for follow-up, including if they wished to meet in their homes or at a neutral location if they preferred other household members not be present during interviews, and how the enumerator should identify themselves if they tried to contact the participant by phone. All participants elected to be interviewed at follow-up visits in their homes. Data collectors introduced themselves as field workers who were raising awareness about maternal and child health, in order to further protect study participants. In both countries, follow-up interviews were conducted with a 2-week delay (e.g., 3.5 months, 6.5 months) in order to allow a buffer for women who needed subsequent doses of injectables.

In Uganda, all baseline survey data were collected using tablets, and questionnaires were available in English and locally spoken languages (Luganda, Lumasaba, Runyankole, Runyoro, Lusoga, and Lugwere). Enumerators were typically fluent in English and at least 1 other language and were assigned to health facilities where the second language was more likely to be used by survey respondents. Follow-up interviews were conducted by mobile phone. Women who did not own a phone at baseline were asked to provide alternate contact phone numbers (e.g., a friend's phone number or the number of a village phone vendor). All women were asked to provide primary, secondary, and alternative phone contacts to be used in case they could not be reached at the primary phone number as well as preferred days and times to be reached. All women who completed the survey were given a small gift of mobile phone airtime, worth 5,000 Ugandan shillings (approximately US$1.40), transferred to the first phone number provided, to compensate her for her participation with the survey. Women received a similar gift upon completion of the follow-up interviews. To follow-up, enumerators attempted to contact the women at the preferred times. If the phone line was busy, women were re-contacted. Excluding busy responses, at least 3 attempts were made to reach each woman via phone. After 3 non-busy attempts, women were considered lost to follow-up.

Following baseline data collection, the government of Uganda implemented a new policy requiring all SIM cards be registered using an individual's national identification card. Non-registered phones were to shut off during the summer of 2017, greatly affecting our ability to follow-up with a potentially large number of women at 3 months. In the 3-month follow-up, all women whose phone line had been switched off or was continuously busy at the first follow-up were sought in person by ProFam agents to see if they were willing to continue to be engaged in the study. If women were identified, agents did not conduct the survey immediately but provided a mobile phone to allow the trained enumerator to collect the 3-month follow-up survey. At the end of the survey, women were asked to provide new phone numbers to be reached for subsequent follow-up surveys.

In both countries, sample size calculations were based upon a hypothesized rate of discontinuation in each setting, with a 95% confidence interval, precision of 0.07, and 80% power. Loss to follow-up, given the 12-month duration of follow-up in this cohort study, and potentially low mobile phone ownership at baseline were important potential factors that we accounted for in the sample size calculations. We planned to assess correlations between measures of observed structural and process quality, as well as the self-reported MII and discontinuation of family planning over the 12-month period. Given the large number of correlations, the analyses needed to account for the increased probability of a false positive. As an approximation of the sample size required to reach a higher type 1 error threshold, the chosen sample size was based upon α=0.01. Given the 2 different contexts, the design effect and assumed loss to follow-up differed for each sample size calculation. In Uganda, we determined that we needed to have at least 530 women complete our endline survey. We also assumed a design effect of 1.5, which generated a minimum number of 796 women completing the survey at 12 months. After inflating this up to account for loss to follow-up (30%), we needed to enroll at least 1,140 eligible women to complete the baseline interview. In Pakistan, a sample of 514 women needed was adjusted by a design effect of 1.3, and 20% potential loss to follow-up, for an effective sample of 800.

### Treatment of Missing Data

All women who were enrolled in the study had complete baseline information. In Pakistan, none of the dates of discontinuation were missing. In Uganda, 12% of women who discontinued while in need had a date of discontinuation which was missing or set to missing due to the fact that the reported date did not fall between 2 adjacent rounds of data collection. These dates were imputed by taking a random date in between the 2 adjacent rounds of data collection for the woman. Women who reported method discontinuation but did not report a reason for discontinuation were assumed to have discontinued while in need. Women were considered lost to follow-up if they could not be located at the address or phone number(s) given, or if they were not available after 3 attempts to contact them.

### Definition of Variables

The MII was calculated by summing the binary responses to the following 3 questions:

1. “During your visit today, were you told about other methods of family planning that you could use?”
2. “Were you told about side effects or problems that you might have with (your chosen) method?”
3. “Were you told what to do if you experienced side effects or problems?”

The Method Information Index sums a client's binary responses to 3 questions on whether the client was told about other methods, side effects, and what to do if she experienced side effects.

The index, ranging from 0 to 3, was used as an ordinal variable as well as a binary variable (3 or less than 3) in the analyses. Age was categorized into 3 groups (15–24, 25–34, 35+), and a woman's primary baseline method was categorized as short- or long-acting (implant or IUD). The household's relative wealth was assessed using an asset index, benchmarked to the most recent DHS survey from each country (2012–2013 in Pakistan, 2016 in Uganda). The asset index was generated from the EquityTool, a shortened list of country-specific assets that are highly correlated with the full list of assets used to generate the wealth index employed by DHS.^29^

Time to discontinuation was treated as a continuous variable, measured in days, with a maximum allowable time of 360 days in Pakistan and 300 days in Uganda. Time in Uganda was truncated due to violation of model assumptions at the end of the reporting period. No events took place in the final 60 days of the reporting period. The event of interest was defined as discontinuation of a modern method while in need. The event occurred if the self-reported reason for discontinuation of any modern method (not necessarily the method obtained at baseline) was method-related (side effects, health concerns, method failure); related to access to resupply (cost, travel time); or social (disapproval of a family member). Women who discontinued for other reasons without switching to another modern method were censored.

### Analytic Methods

This study used survival analysis and Cox proportional hazard models with robust standard errors to account for clustering by facility to assess the degree of correlation between MII and discontinuation. Log-rank tests and Kaplan-Meier survival curves assessed the unadjusted effect of MII. Discontinuation rates were estimated from survival curves. Explanatory variables tested in each country were age, wealth group, parity, education, method type at baseline (short- or long-acting), and user type at baseline (first-time user, returning to contraception after a lapse in use, method switcher). Assumptions of proportionality for each covariate were tested numerically and graphically. We tested correlation between the potential covariates and dropped parity due to a high correlation with age. Variables were considered for the final adjusted model if they were significant at *P*≤.10 when included with MII in a Cox proportional hazard model. We tested each significant variable to see if it was time-dependent and assessed model fit using parameters available in Stata 13.^30^^(pp164-194)^ In Uganda, a model curtailed at 300 days was compared to one for the full available time, and the curtailed model had better fit without changing model parameters. Finally, we tested the significance of the joint effect of method selected at baseline and MII on discontinuation. The parsimonious Cox proportional hazard model is thus presented for both contexts, adjusted for covariates that met significance criteria (*P*≤.10) in at least 1 country. The results are presented in the form of crude and adjusted hazard ratios; the adjustment accounts for women's age category, prior contraceptive use (new user, switcher, lapsed user), and whether a short- or long-acting method was adopted at baseline.

### Ethical Approval

Approval for the study arm in Pakistan was obtained from Ethical Review Committee (ERC) Marie Stopes International (MSI), UK (022-16), and the National Bioethics Committee (NBC) at Pakistan Medical Research Council (PMRC), Islamabad (4-87/17/NBC-227/RDC/2308). Approval for the study arm in Uganda was obtained from the Makerere University School of Public Health Higher Degrees Research and Ethics Committee (451) and the Uganda National Council of Science and Technology (UNCST), Kampala (SS4215).

## RESULTS

**FIGURE 1:**
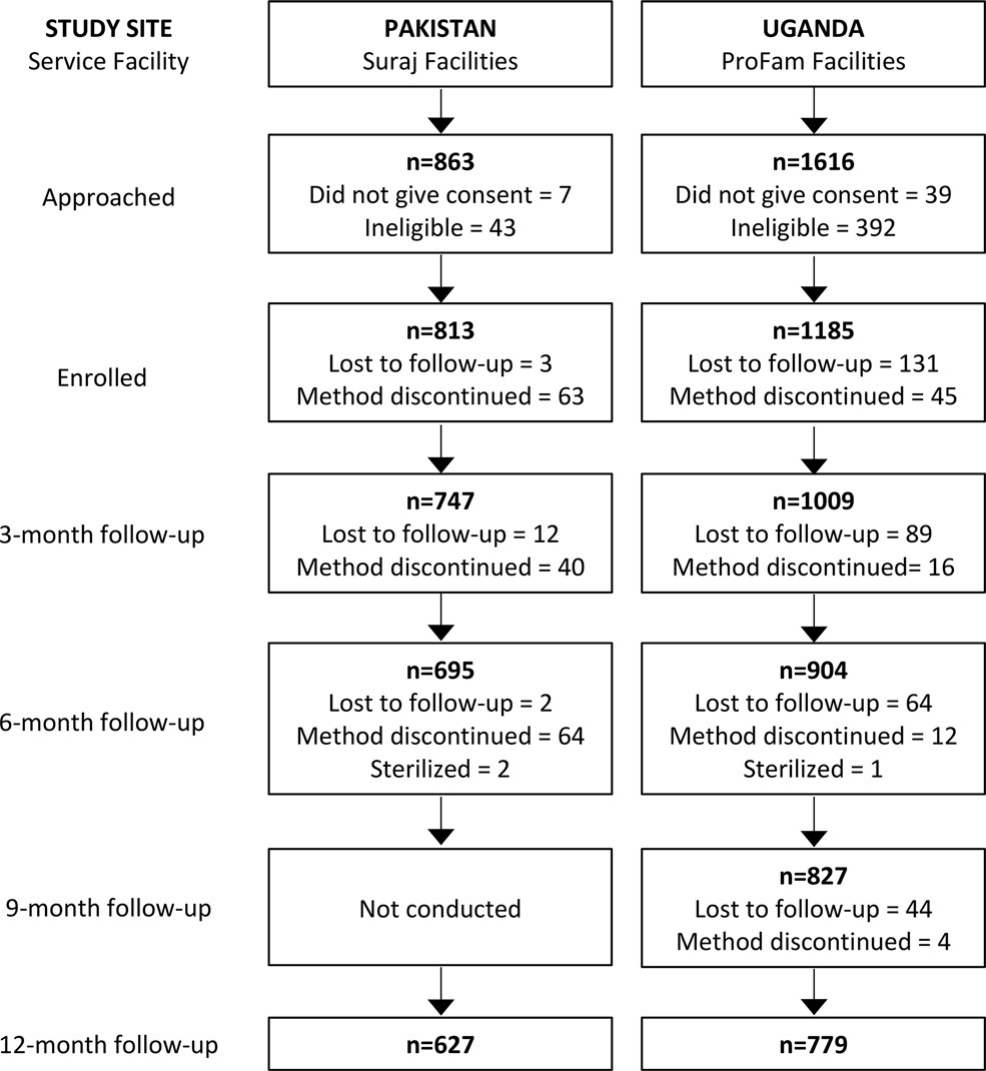
Flow Diagram of Participant Enrollment and Follow-Up Note: Women who were lost to follow-up are indicated in the flow chart at the time they were last contacted, while those who discontinued between 2 rounds are shown in the former round.

A total of 1,998 women were enrolled across the 2 countries: 813 women from 75 Suraj facilities in Pakistan and 1,185 women from 30 ProFam facilities in Uganda (Figure 1). Of those enrolled, 2.1% (n=17) in Pakistan and 27.7% (n=328) in Uganda were lost to follow-up.

Table 1 summarizes the demographic and reproductive characteristics of study participants, according to country. The sample comprised relatively younger women in Uganda (40% under 24 years of age) compared with Pakistan (15% under 24 years of age). As a result, women in the sample from Uganda reported lower parity (70% reported 3 or fewer live births) compared with Pakistan (52% reported 3 or fewer live births). Women in the sample from Uganda were also more likely to have received education (only 2.4% never having gone to school) compared with Pakistan (57.6% reported never having gone to school). Three-fourths of the sample in Uganda belonged to the highest (wealthiest) quintile. In contrast, 47.9% of the women in the sample from Pakistan belonged to middle or lower wealth quintiles.

**TABLE 1.** Baseline Characteristics of Study Participants, by Country

	Pakistan (n=813) No. (%)	Uganda (n=1185) No. (%)
**Age group, years**		
15–24	125 (15.4)	475 (40.1)
25–34	443 (54.5)	531 (44.8)
35–49	245 (30.1)	179 (15.1)
**No. of prior live births**		
None	1 (0.1)	112 (9.5)
1	97 (11.9)	255 (21.5)
2–3	317 (39.0)	448 (37.8)
4–5	235 (28.9)	239 (20.2)
6 or more	163 (20.0)	131 (11.1)
**Highest completed education**		
None (never went to school)	468 (57.6)	29 (2.4)
Primary	145 (17.8)	401 (33.8)
Secondary	151 (18.6)	595 (50.2)
Beyond secondary	49 (6.0)	160 (13.5)
**Wealth quintile**		
1 (lowest)	54 (6.6)	21 (1.8)
2	126 (15.5)	46 (3.9)
3	210 (25.8)	43 (3.6)
4	228 (28.0)	180 (15.2)
5 (highest)	195 (24.0)	895 (75.5)
**User type**		
First-time adopter	294 (36.2)	314 (26.5)
Lapsed user	42 (5.2)	177 (14.9)
Switcher	477 (58.7)	694 (58.6)
**Type of method adopted at baseline**		
Intrauterine device	350 (43.1)	276 (23.3)
Implant	0 (0.0)	431 (36.4)
Injectable	199 (24.5)	335 (28.3)
Pill	149 (18.3)	122 (10.3)
Male condom^a^	115 (14.2)	21 (1.8)

a Also includes 1 female condom user and 1 emergency contraceptive user in Uganda.

Of those enrolled, approximately 3 in 5 women across both countries who left with a modern method were using a different contraceptive method when they came to the clinic (switcher).In contrast, 36.2% in Pakistan and 26.5% of women in Uganda were first-time adopters of a contraceptive method. More women in Pakistan (56.9%) chose a short-acting method at baseline, notably driven by use of condoms, as opposed to Uganda where 59.7% chose an implant or IUD.

**FIGURE 2:**
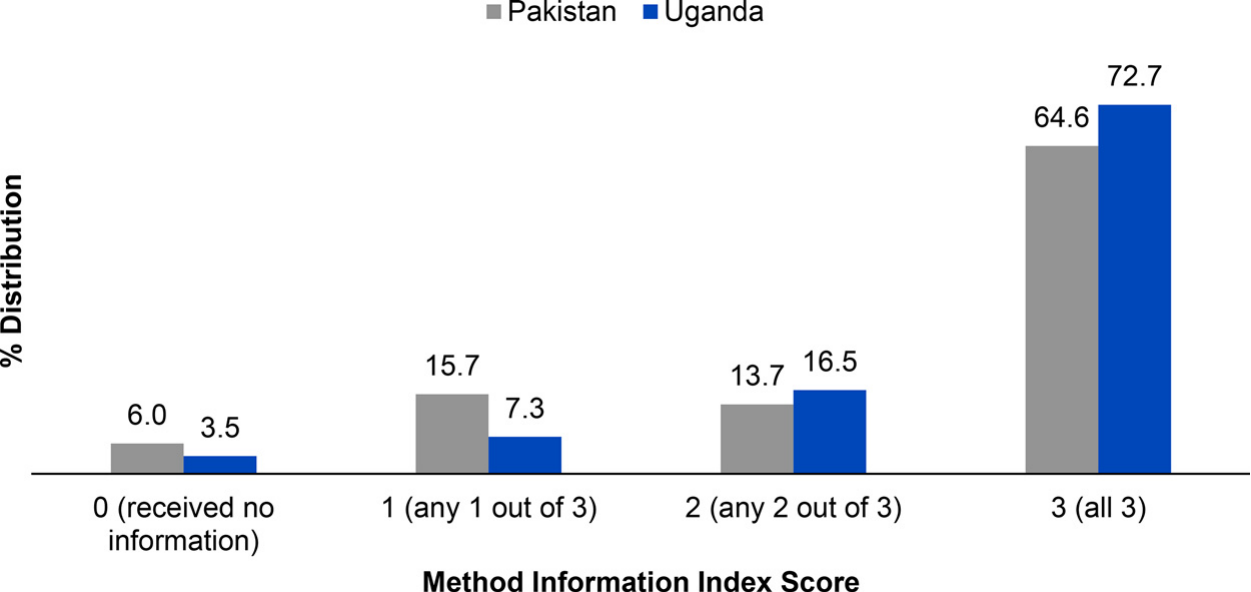
Distribution of Method Information Index Scores, by Country

Figure 2 presents the distribution of MII scores according to country. At baseline, 64.6% of women in Pakistan and 72.7% in Uganda reported receiving information about all 3 MII aspects from their service provider when they began using their method.

At baseline, 65% and 73% of women in Pakistan and Uganda, respectively, reported receiving information about all 3 aspects of the Method Information Index.

**FIGURE 3:**
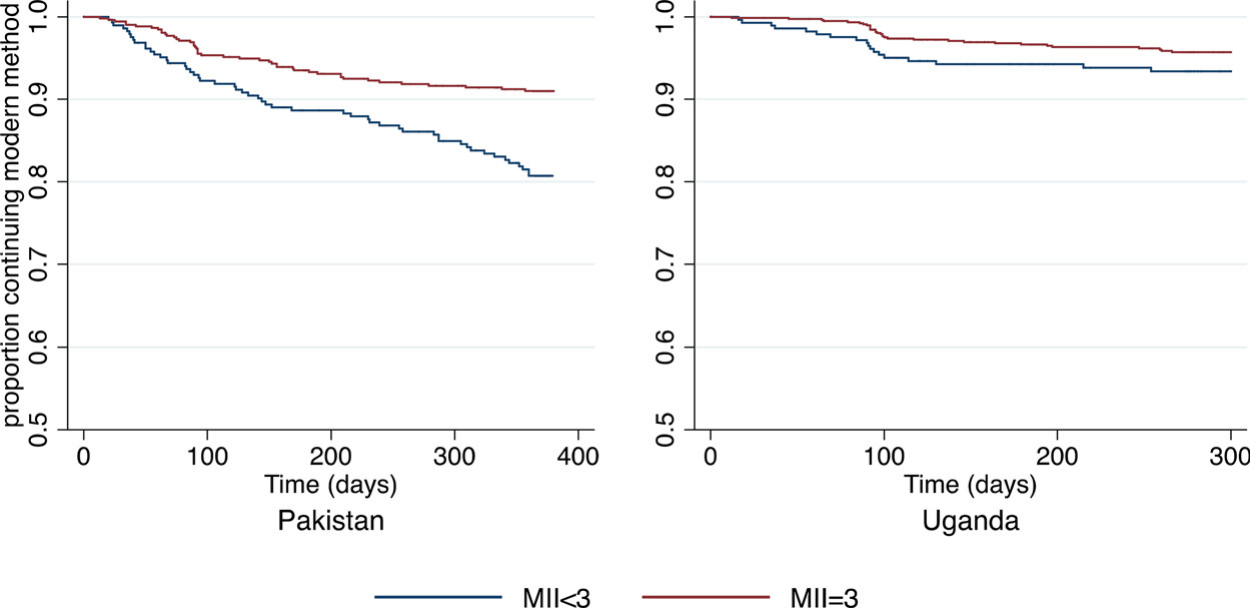
Cumulative Probability of Modern Method Continuation Among Women in Need, by MII Score (Binary) and Country Abbreviation: MII, Method Information Index.

**FIGURE 4:**
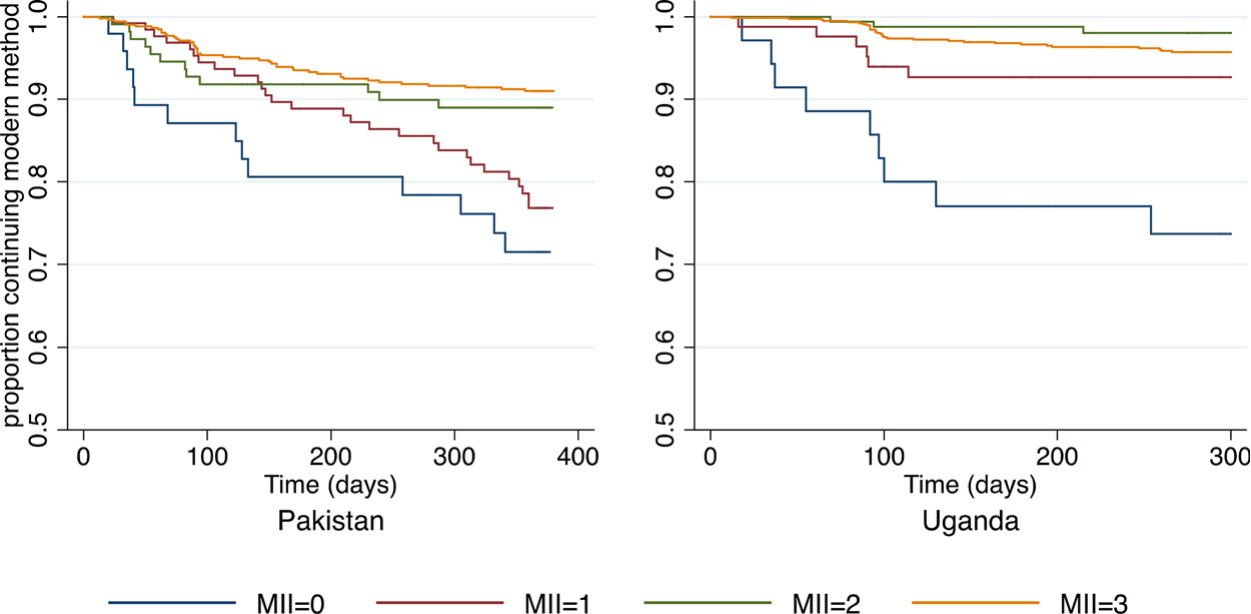
Cumulative Probability of Modern Method Continuation Among Women in Need, by MII Score (Ordinal) and Country Abbreviation: MII, Method Information Index.

In Pakistan, among the 165 women who stopped using modern methods, 98 (59.4%) discontinued while in need. In Uganda, of the 77 women who stopped modern method use, 50 (64.9%) discontinued while need. In Figure 3 and Figure 4, Kaplan-Meier survival curves are used to compare the probabilities of continuation of a modern method over the 12-month study period. Women who discontinued a modern method because they no longer had need were censored, and time is reported in days. Figure 3 compares the cumulative probability of women continuing to use their modern method between those who received information about all 3 MII aspects (MII=3) and those who received less information (MII<3). In Pakistan, the continuation rates differed significantly between the 2 groups; by 360 days, the probability of continuation was 0.91 for women with an MII score of 3 versus 0.81 for those with a score of less than 3 (*P*<.001). A similar trend was observed in Uganda; however, this difference was not statistically significant (log-rank test *P* value=.10). The cumulative probability of method continuation when MII is stratified by score (ranging from 0 to 3) is presented in Figure 4. In Pakistan, we observed an incremental improvement in continuation rates with a unit increase in MII scores. The 12-month probability of method continuation among women who had a MII of 0, 1, 2, and 3 were 0.72, 0.77, 0.89, and 0.91, respectively, and the log-rank test of equality indicated that the curves were significantly different (*P*<.001). In Uganda, the 4 curves were also significantly different from each other (*P*<.001) and the lowest rate of continuation (0.78) was found among women who received no information about any aspect of MII. The 12-month probability of continuation did not differ substantially between other MII groups (MII=1, 0.93; MII=2, 0.98; and MII=3, 0.96).

Table 2 and Table 3 show the risks of contraceptive discontinuation while in need, associated with binary and ordinal measures of MII, respectively. In Pakistan, the risk of method discontinuation among women who were informed about all aspects of MII (MII=3) decreased by 48% (adjusted hazard ratio [HR_adj_]=0.52; 95% confidence interval [CI]=0.32 to 0.85; *P*=.009) compared with women who were not told about all aspects of MII (MII<3). Although the direction of the relationship was similar, neither the crude nor the adjusted model demonstrated a statistically significant effect of the binary measure of MII on method discontinuation for the sample from Uganda (HR_adj_=0.64; 95% CI=0.35 to 1.18; *P*=.16). In both countries, women who obtained a short-acting method at the baseline visit were significantly more likely to discontinue while in need. In neither country did adjustment for this, and other variables, affect the magnitude or significance of the MII to discontinuation relationship, seen by comparing the crude and adjusted hazard ratios.

**TABLE 2.** Risk of Modern Method Discontinuation While in Need, by Country, With MII as a Binary Variable

	Pakistan (N=810)				Uganda (N=1,054)			
	Unadjusted HR (95% CI)	*P* Value	Adjusted HR (95% CI)	*P* Value	Unadjusted HR (95% CI)	*P* Value	Adjusted HR (95% CI)	*P* Value
**MII Score**								
MII<3 (ref.)	–		–		–		–	
MII=3	0.45 (0.28, 0.74)	.001	0.52 (0.32, 0.85)	.009	0.62 (0.35, 1.09)	.097	0.64 (0.35, 1.19)	.16
**Type of Method Used at Baseline**								
LARC (ref.)								
Short-acting method			1.75 (1.10, 2.80)	.02			7.67 (3.76, 15.63)	<.001
**Age Group, years**								
35–49 (ref.)								
15–24			1.43 (0.74, 2.75)	.28			2.06 (0.69, 6.13)	.19
25–34			1.56 (0.91, 2.68)	.11			2.52 (1.04, 6.13)	.04
**Prior Contraceptive Use**								
First-time adopter (ref.)								
Return user			0.73 (0.25, 2.12)	.56			1.86 (0.73, 4.74)	.19
Method switcher			0.63 (0.43, 0.91)	.02			1.09 (0.50, 2.39)	.83

Abbreviations: CI, confidence interval; HR, hazard ratio; LARC, long-acting reversible contraceptive; MII, Method Information Index.

**TABLE 3.** Risk of Modern Method Discontinuation While in Need, by Country, With MII as an Ordinal Variable

	Pakistan (N=810)				Uganda (N=1,054)			
	Unadjusted HR (95% CI)	*P* Value	Adjusted HR (95% CI)	*P* Value	Unadjusted HR (95% CI)	*P* Value	Adjusted HR (95% CI)	*P* Value
**MII Score**								
0 (ref.)								
1	0.73 (0.29, 1.82)	.50	0.73 (0.29, 1.84)	.51	0.25 (0.09, 0.74)	.01	0.32 (0.12, 0.83)	.02
2	0.35 (0.14, 0.90)	.03	0.48 (0.16, 1.42)	.18	0.06 (0.02, 0.22)	<.001	0.10 (0.03, 0.34)	<.001
3	0.28 (0.11, 0.70)	.007	0.35 (0.13, 0.95)	.04	0.14 (0.07, 0.29)	<.001	0.19 (0.08, 0.44)	<.001
**Type of Method Used at Baseline**								
LARC (ref.)								
Short-acting method			1.53 (0.86, 2.71)	.15			6.79 (3.41, 13.52)	<.001
**Age Group, years**								
35–49 (ref.)								
15–24			1.40 (0.73, 2.70)	.32			2.36 (0.78, 7.19)	.13
25–34			1.54 (0.90, 2.64)	.12			2.71 (1.05, 6.96)	.04
**Prior Contraceptive Use**								
First-time adopter (ref.)								
Return user			0.75 (0.26, 2.21)	.61			1.73 (0.64, 4.69)	.28
Method switcher			0.65 (0.44, 0.96)	.03			1.03 (0.47, 2.21)	.95

Abbreviations: CI, confidence interval; HR, hazard ratio; LARC, long-acting reversible contraceptive; MII, Method Information Index.

**FIGURE 5:**
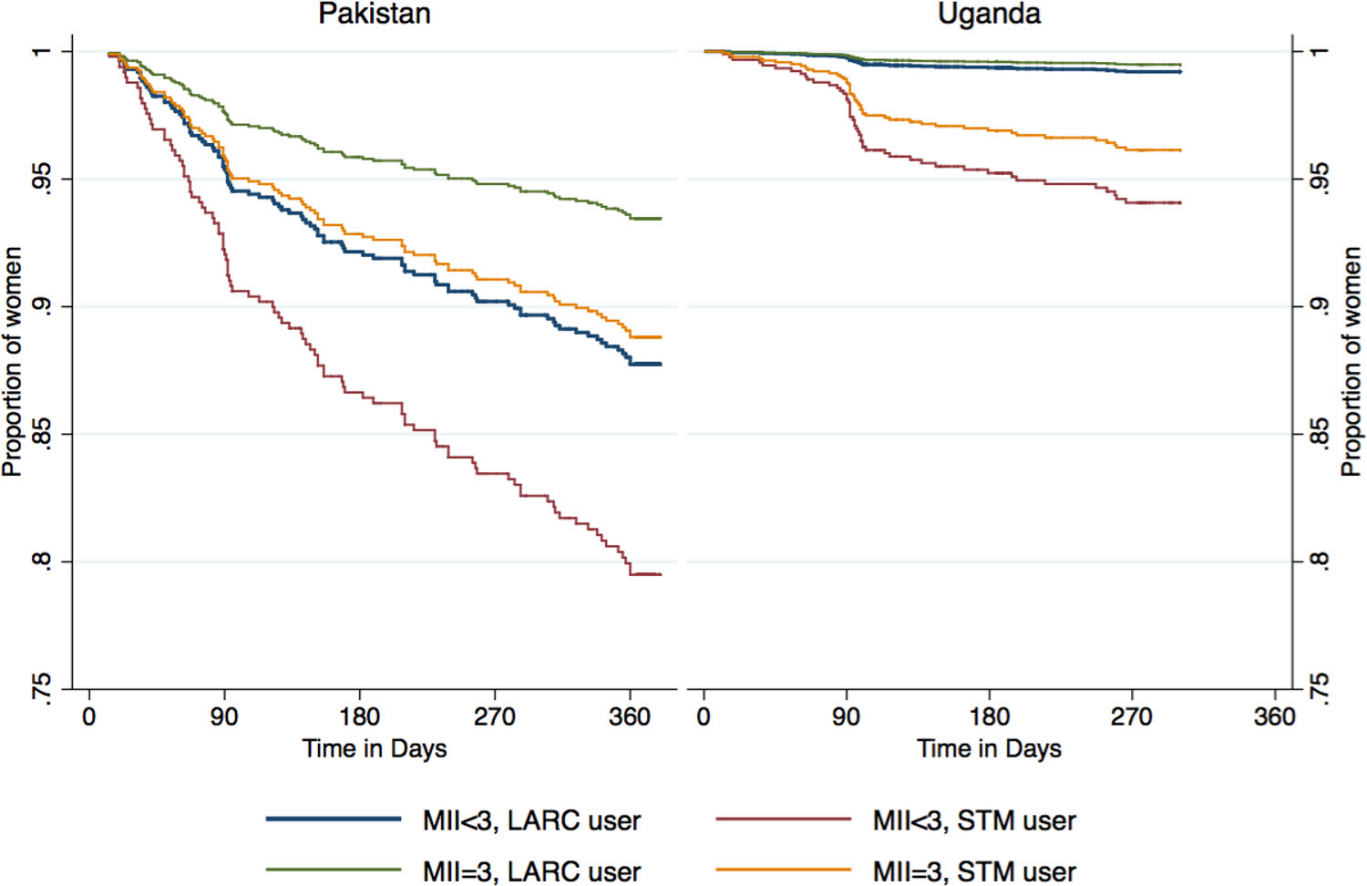
Cumulative Probability of Modern Method Continuation Among Women in Need, by MII Score (Binary) and Method Type Used at Baseline, by Country Abbreviations: LARC, long-acting reversible contraceptive; MII, Method Information Index; STM, short-term. Note: Model presented for new users aged 35–49 years.

Figure 5 depicts the combined effect of MII score and method type used at baseline on method continuation. The blue line, representing women who reported receiving less than 3 pieces of information and who obtained a long-acting method at baseline, is the same as the baseline hazard for the overall Cox proportional hazards model. In Uganda, there was no significant difference in method continuation between users who received full counseling information and those who received incomplete information (regardless of the type of method used at baseline). A comparison of the blue line (MII<3, LARC user) and green line (MII=3, LARC user) for Pakistan shows an approximately 6% significant difference in the proportion of women who used a LARC at baseline continuing method use at 12 months. In other words, women starting a LARC method who did not receive full counseling information were more likely to discontinue at 12 months than women receiving a LARC method and full counseling information. Visually, this difference appears more pronounced in Pakistan when comparing short-acting method users (orange vs. red lines). To investigate if the method type received at baseline differentially affected the relationship between MII score and discontinuation, we tested the joint effect of these 2 variables. The joint effect, i.e. the difference in the 2 differences (blue vs. green lines versus orange vs. red lines), was not significant in either country, and likelihood ratio tests indicated that the adjusted models presented in Table 2 were not significantly different than a model with the joint effect (results not shown). Therefore, the effect of MII score on discontinuation did not differ among short- versus long-acting method users.

In both countries, we found that as the ordinal MII score increased, the risk of discontinuation while in need decreased (Table 3). In Pakistan, the risk of contraceptive discontinuation was 65% lower (HR_crude_=0.35; 95% CI=0.14 to 0.90; *P*=.03), and 72% lower (HR_crude_=0.28; 95% CI=0.11 to 0.70; *P*=.007), among women who were told about any 2, or any 3 aspects of MII, respectively, than among women not told about any aspects of MII. In the adjusted model, however, only the difference in the risk of contraceptive discontinuation between MII=3 and MII=0 remained statistically significant (HR_adj_=0.35; 95% CI=0.13 to 0.95; *P*=.04). Significant differences in risk of method discontinuation were also seen between women who reported MII=1 and MII=3 (*P*=.006); there were no significant differences in risks of discontinuation between MII=1 versus MII=2, and between MII=2 versus MII=3 (results not shown).

In both countries, as the ordinal Method Information Index score increased, the risk of discontinuation while in need decreased.

In Uganda, the ordinal measure of MII exhibited strong association with contraceptive discontinuation: women who reported being informed about all aspects of MII were 80% less likely to discontinue while in need (HR_adj_=0.19; 95% CI=0.08 to 0.44; *P*<.001), women informed about any 2 aspects of MII were 90% less likely (HR_adj_=0.10; 95% CI=0.03 to 0.34; *P*<.001), and women who were informed about any 1 aspect of MII were 68% less likely (HR_adj_=0.32; 95% CI=0.12 to 0.83; *P*=.02) to discontinue contraceptive use while in need as compared to women who reported not being informed about any aspect of MII. Moreover, in Uganda, risk of method discontinuation was significantly lower for women with an MII score of 2 versus 1 (*P*=.03). There was no significant difference in hazard of method discontinuation between MII=1 versus MII=3, and between MII=2 versus MII=3 (results not shown).

Table 4 shows the effect of each MII aspect (question) on discontinuation of modern contraception while in need, according to the country. In the unadjusted model, each MII question is considered separately, while in the adjusted model, all 3 questions are included, in addition to women's age category, prior contraceptive use category, and choosing a short-acting method at baseline. In Pakistan, the crude estimates show that provision of information to women about potential side effects (HR_crude_=0.36; 95% CI=0.24 to 0.54; *P*<.001), and what to do if they occur (HR_crude_=0.36; 95% CI=0.24 to 0.54; *P*<.001), at the time of method adoption decreased the likelihood of method discontinuation. However, the relationship became insignificant when adjusted for the other MII questions and the covariates. In Uganda, the chances of method discontinuation significantly dropped (73% lower risk of discontinuation) when women were informed about other methods (HR_adj_=0.27; 95% CI=0.13 to 0.56; *P*<.001), and when they reported being informed about how to manage side effects if they occur (55% lower risk of discontinuation; HR_adj_=0.45; 95% CI=0.24 to 0.85; *P*=.01).

**TABLE 4.** Risk of Modern Method Discontinuation While in Need, by MII Aspect (Question), by Country

MII Questions	Pakistan				Uganda			
	Unadjusted HR (95% CI)	*P* Value	Adjusted HR^a^ (95% CI)	*P* Value	Unadjusted HR (95% CI)	*P* Value	Adjusted HR^a^ (95% CI)	*P* Value
Informed about other methods (ref.=no)	0.74 (0.45, 1.21)	.23	0.89 (0.44, 1.82)	.75	0.27 (0.14, 0.50)	<.001	0.27 (0.13, 0.56)	<.001
Informed about side effects (ref.=no)	0.36 (0.24, 0.54)	<.001	0.58 (0.32, 1.07)	.08	0.61 (0.33, 1.14)	.12	1.74 (0.72, 4.22)	.22
Informed of what to do if experienced side effects (ref.=no)	0.36 (0.24, 0.54)	<.001	0.73 (0.41, 1.31)	.29	0.3 (0.16, 0.55)	<.001	0.45 (0.24, 0.85)	.01

Abbreviations: CI, confidence interval; HR, hazard ratio; MII, Method Information Index.

a Adjusted for participants' age, prior contraceptive use, and short-acting baseline method use. All 3 MII questions are included in the adjusted model.

## DISCUSSION

High-quality family planning counseling, as measured by the MII score at clinic exit, was associated with subsequently higher 12-month continuation rates in this clinic-based prospective cohort study. The main findings are consistent with the literature, which posits that a client's receipt of contraceptive information is a critical component in realizing full method choice and high-quality care.^8^ Although other studies have found that a facility's quality of care is associated with subsequent continuation of contraceptive use,^12^^,^^31^^,^^32^ to the best of our knowledge, this is the first study to find a persistent pattern of high MII scores at facility exit and lower rates of discontinuation over a 12-month follow-up period. Looking at the MII as an ordinal variable, the largest difference is between receiving no information and receiving any 1 of the 3 pieces of the MII, suggesting that at the very core, any counseling (versus nothing) matters. While the MII has traditionally been presented as binary (receiving all 3 pieces, or not receiving all 3 pieces of information), our findings suggest value in looking at the MII as a more conventional index.

High-quality family planning counseling, as measured by the client-reported Method Information Index at clinic exit, was associated with subsequently higher 12-month continuation rates.

Although other studies have looked at aspects of counseling, counseling interventions, or other correlates of discontinuation, this is the first to our knowledge to look specifically at the MII. As a key indicator of FP2020, the MII is increasingly a standard indicator for family planning programs. The association between the MII and method continuation found in this study is promising as it demonstrates that the MII can be measured at the clinic level and be comparable across clinics and networks of clinics.

Our findings suggest that the Method Information Index can be measured at the clinic level and be comparable across clinics and networks of clinics.

Our study also finds that the type of method obtained at baseline (short- vs. long-acting) is significantly associated with discontinuation while in need, unsurprising given that method-related dissatisfaction is the most common reason for method discontinuation globally.^3^^,^^6^^,^^33^ However, comprehensive counseling, as measured by higher MII scores, is associated with improved continuation, irrespective of method type chosen. Which, if any, single aspect of counseling, may influence this relationship is not clear from our analyses. In Pakistan, where women experienced higher rates of discontinuation, no single MII question was more strongly protective of discontinuation, while in Uganda, being informed of other methods or how to deal with side effects mattered more than being told about the side effects. Abdel-Tawab and RamaRao describe the relationship between client-provider interaction and contraceptive continuation as a puzzle, to which our study contributes a piece. The MII is a way to assess the information-giving component of the client-provider interaction, but as the authors note, it does not capture non-verbal communication, empathy, or partnership building.^34^ The index also does not capture the content of information exchanged. In related research with this same population, we used follow-up questions to “adjust” the MII score, reducing the score if the side effects reported by the client, for example, were not medically associated with the method she received. Depending on the strictness of the definition, adjusted and unadjusted MII scores were significantly different.^16^ Nevertheless, to the extent that the information offered by the provider is a necessary factor in plugging the “leaky bucket,”^35^ MII is a simple and straightforward assessment.

### Strengths and Limitations

Despite the significance of the study, there are several limitations to note. Given that the sample of facilities in both Pakistan and Uganda is drawn from social franchise networks, which tend to be more urban, serve middle-income clients, and often receive both family planning training and supplies directly from the organizations/franchisors who sponsor them, care should be taken in generalizing findings beyond those networks.^36^ Further research is needed to understand if similar associations are present in national (public-sector) programs, where counseling may not be as strongly emphasized.

The reliance on mobile phones for follow-up and the introduction of the policy to switch off non-registered phones in Uganda may have also affected our ability to follow-up with women and may have affected the comparability of the 2 countries. Researchers designed follow-up interviews in Uganda to be conducted by mobile phone, a decision made in deference to the operational practicality, low unit costs, and high mobile phone ownership in Uganda. The researchers anticipated a high loss to follow-up in their sample size calculations. However, there may have been unbalanced loss to follow-up by country. The factors associated with the participant being unable or unwilling to continue participating in the study (e.g., lack the means to maintain her phone) may be similar to the factors associated with contraceptive discontinuation (e.g., lack the means to return to facility for pill refills or injections). Since the analysis truncated data from participants lost to follow-up, any discontinuation they experienced later would be unobserved. Compared to the Pakistan cohort, differences in discontinuation rates between low and high MII participants in the Uganda cohort was less pronounced; however, method discontinuation rates may have been confounded by access to phones in Uganda and the analysis may have underestimated the relationship between the MII and method discontinuation.

Another potential limitation of this study is the response bias inherent to MII. Clients are self-reporting what they recall being told about key components of the contraceptive counseling process. In the absence of observation of the actual counseling session, this analysis relies on self-reported data. Prior work comparing exit interview data to observation in facility surveys demonstrated that clients tended to overreport when asked if they received counseling about side effects of their method.^37^^,^^38^ However, the impact of this limitation is unclear as there is no evidence to suggest that what women perceive they were told during their counseling session is any less salient or significant in terms of subsequent contraceptive use.^39^

Finally, the analyses presented here limit the assessment of quality to the MII. In reality, quality is a multidimensional construct,^7^ and the facilities and providers who do a better job at counseling may also perform better on other aspects of quality, such as their technical competence, availability of methods, or general patient-provider interaction. Testing the relationship of facility quality and MII simultaneously on discontinuation would have required a far larger sample of facilities; this may be an area of further research.

The study design also has several unique strengths. First, in both settings, the study collected a robust amount of data over 12 months, including contraceptive continuation over time. The study likely captured the majority of contraceptive discontinuation as the highest rates of discontinuation tend to occur within the first 12 months.^3^^,^^40^ Second, the MII questions are asked of clients as part of an exit survey, minimizing recall bias for this measure, which often has been asked retrospectively of clients in population-based surveys, sometimes months or years after the service. Finally, this study was implemented in 2 different settings, where the sample in Pakistan included fewer youth, more participants with no schooling, and more who belonged to the middle or lower wealth quintiles compared with Uganda. We obtained similar findings in both of these settings, however.

Importantly for programs, the MII provides a measure of quality that is highly implementable and relevant to important outcomes such as contraceptive continuation. The short, streamlined nature of the index is desirable for programs and makes the measure appealing and scalable as opposed to other indices composed of a long list of questions that require more time for clients to respond to and are easier to implement incorrectly. Social franchises can undertake this simplified measure much as they have adopted the EquityTool. Additionally, given the 3-question simplicity of the MII, routine monitoring of family planning quality may be possible using mobile technology that engages consumers in post-service accountability.

Studies on the impact of MII provide evidence that further justify the use of MII in establishing and monitoring policy objectives. For FP2020, the MII is included among the 18 core indicators as a key quality metric to track progress in meeting the goal of 120 million additional contraceptive users by 2020. Greater adoption of MII across governments' reporting systems brings with it the potential to manage quality improvement initiatives, set standards for minimum quality, and identify reasonable targets in the next generation of strategic purchasing initiatives.^41^^,^^42^ For example, the MII is being explored as a quality metric to link to incentives in results-based financing initiatives funded by the Global Financing Facility (GFF) and partner governments. The MII is an important opportunity to link family planning quality to purchasing mechanisms that will increasingly draw from domestic resources over the near future.^43^^–^^45^

## CONCLUSION

Our study found a positive association between higher MII, collected from exit interviews with family planning adopters, and method continuation over 12 months in a sample of clients of social franchises from 2 diverse settings. While future work is needed to better understand whether this relationship holds in public-sector facilities, our findings are important because while MII has been adopted widely as an indicator for systems quality, this study provides the first strong evidence of the value of MII as a validated measure of facility quality. Management of the use of MII is facilitated by regular collection of data that correlate to better performance. The short, easy-to-collect nature of the MII and the validation of a link between index performance and improved outcomes therefore has important programmatic implications. Use of MII at the program and point-of-service level may facilitate more feasible, routine measurement of quality and more impactful actions to assure and improve quality in resource-strained facilities. Our findings require replication in other settings but, if confirmed, will have important programmatic and policy implications for service delivery and regulatory frameworks. A validated tool to assess information exchange in a family planning counseling session, a key component of family planning service quality, offers an important opportunity to monitor, benchmark, compare, and improve programs that provide family planning services, and through this to positively impact reproductive outcomes.

Additional research is needed to better understand whether the association between the Method Information Index and contraceptive continuation holds in public-sector facilities.

## Notes

**Peer Reviewed**

**First Published Online:** March 7, 2019

**Funding:** Funding for the study arm in Pakistan was provided by the United States Agency for International Development (USAID) through its Sustaining Health Outcomes of the Private Sector (SHOPS) Plus Project, while funding support for the social franchise clinics enrolled in Pakistan was provided by the UKAID Department for International Development (DFID). The Support for International Family Planning Organizations 2 (PSI-SIFPO2) project funded by USAID provides support to both PACE/Uganda and to this study in Uganda.
